# The Sodium Content of Processed Foods in South Africa during the Introduction of Mandatory Sodium Limits

**DOI:** 10.3390/nu9040404

**Published:** 2017-04-20

**Authors:** Sanne A. E. Peters, Elizabeth Dunford, Lisa J. Ware, Teresa Harris, Adele Walker, Mariaan Wicks, Tertia van Zyl, Bianca Swanepoel, Karen E. Charlton, Mark Woodward, Jacqui Webster, Bruce Neal

**Affiliations:** 1 The George Institute for Global Health, University of Oxford, Oxford OX1 3QX, UK; markw@georgeinstitute.org.au; 2 Carolina Population Center, University of North Carolina, Chapel Hill, NC 27516, USA; edunford@georgeinstitute.org.au; 3 The George Institute for Global Health, University of Sydney, Sydney, NSW 2050, Australia; jwebster@georgeinstitute.org.au (J.W.); bneal@georgeinstitute.org.au (B.N.); 4 Hypertension in Africa Research Team, North West University, Potchefstroom 2520, South Africa; lisa.ware@nwu.ac.za; 5 Discovery Vitality, Sandton 2146, South Africa; terryh@discovery.co.za (T.H.); adelewa@discovery.co.za (A.W.); 6 Center of Excellence for Nutrition, North West University, Potchefstroom 2520, South Africa; 13009494@nwu.ac.za (M.W.); tertia.vanzyl@nwu.ac.za (T.v.Z.); biancaswanepoel.nwu@gmail.com (B.S.); 7 School of Medicine, University of Wollongong, Wollongong, NSW 2522, Australia; karenc@uow.edu.au; 8 Department of Epidemiology, Johns Hopkins University, Baltimore, MD 21218, USA; 9 The Charles Perkins Centre, University of Sydney, Sydney, NSW 2006, Australia; 10 Royal Prince Alfred Hospital, Sydney, NSW 2050, Australia; 11 Imperial College London, London SW7 2AZ, UK

**Keywords:** salt intake, sodium legislation, South Africa, packaged food, nutritional composition

## Abstract

Background: In June 2016, the Republic of South Africa introduced legislation for mandatory limits for the upper sodium content permitted in a wide range of processed foods. We assessed the sodium levels of packaged foods in South Africa during the one-year period leading up to the mandatory implementation date of the legislation. Methods: Data on the nutritional composition of packaged foods was obtained from nutrition information panels on food labels through both in-store surveys and crowdsourcing by users of the HealthyFood Switch mobile phone app between June 2015 and August 2016. Summary sodium levels were calculated for 15 food categories, including the 13 categories covered by the sodium legislation. The percentage of foods that met the government’s 2016 sodium limits was also calculated. Results: 11,065 processed food items were included in the analyses; 1851 of these were subject to the sodium legislation. Overall, 67% of targeted foods had a sodium level at or below the legislated limit. Categories with the lowest percentage of foods that met legislated limits were bread (27%), potato crisps (41%), salt and vinegar flavoured snacks (42%), and raw processed sausages (45%). About half (49%) of targeted foods not meeting the legislated limits were less than 25% above the maximum sodium level. Conclusion: Sodium levels in two-thirds of foods covered by the South African sodium legislation were at or below the permitted upper levels at the mandatory implementation date of the legislation and many more were close to the limit. The South African food industry has an excellent opportunity to rapidly meet the legislated requirements.

## 1. Introduction

Excess dietary salt intake is associated with elevated blood pressure, a major risk factor for cardiovascular diseases [1,2]. In 2010, an estimated 1.65 million cardiovascular deaths worldwide—or 1 out of every 10 cardiovascular deaths—were attributed to salt consumption above the World Health Organization (WHO) recommended intake of 5 g per day [3,4]. Salt reduction has been described by the WHO as one of the best investments to improve public health and an efficient and cost-effective way to decrease the burden of elevated blood pressure and cardiovascular diseases [5].

In 2013, WHO Member States adopted the global target of a 30% reduction of mean population intake of salt by 2025, as part of a broader set of strategies to reduce premature mortality from non-communicable diseases by 25% in 2025 [6]. A growing number of countries are developing and implementing strategies to reduce salt intake, including, but not limited to, food supply reformulations, front of package labelling, taxation, consumer education, and interventions in public institutions [7,8]. For many countries, these strategies are voluntary or restricted to a limited number of food products [9].

The Republic of South Africa was the first country globally to develop comprehensive, mandatory legislation to reduce sodium levels across a wide range of processed food categories, which involved the co-operation of many food industry members from various sectors [10,11]. It is estimated that about half of daily salt intake in South Africa derives from processed foods, with bread being the greatest contributor to non-discretionary salt intake [12,13]. The South African sodium legislation was passed by the Department of Health in 2013 and set restrictions regarding the maximum levels of sodium allowed in several commonly consumed foods which, in addition to bread, include breakfast cereals, margarines, meat products, snack foods, and soup mixes [10]. A few products that are high in sodium, such as biltong (“jerky”) and soy sauce, were exempted due to their relatively low contribution to sodium in the South African diet. The legislation aims to reduce the amount of sodium in specific foods in two waves; the first came into force in June 2016 and the second, with lower sodium targets, will come into effect in June 2019. If successful, this new strategy to reduce sodium in the food supply is expected to save thousands of lives annually and to yield substantial cost savings to the South African health service [14,15].

To measure progress in reducing the sodium levels of foods, identify challenges, and track changes over time, an assessment of the current sodium levels of processed foods in South Africa is needed. In the present study, we used data from nutrition information panels on food labels to evaluate the sodium levels of packaged foods in South Africa during the one-year period leading up to the implementation date for the legislation.

## 2. Methods

### 2.1. Data Sources

A database with information on the nutritional composition of packaged foods available for consumer purchase in South Africa was established through in-store surveys and crowdsourcing of food labels by users of the HealthyFood Switch mobile phone app [16]. Store surveys were done through collaboration with Discovery, South Africa’s largest private health insurance company. Part of Discovery’s health promotion programme is Vitality, which partners with selected South African retailers to offer the HealthyFood benefit [17]. Researchers visited major South African retail stores in Johannesburg, including Woolworths, Pick n Pay, Spar, and Shoprite Checkers, and took photos of all packaged food and beverage items using The George Institute’s Data Collector smartphone application and the HealthyFood Switch smartphone application [18]. These applications enable the user to scan the barcode of a packaged food item, and then take multiple photographs of the item to capture the product name, nutritional information, and ingredient list. These data are then used to populate a database from which the HealthyFood Switch smartphone app draws information. Consumers can use this app to scan the barcodes of packaged foods using their smartphone camera, which will then display on-screen, easy-to-interpret nutritional information along with suggestions for similar, but healthier, alternative products. When a product is not present in the database, the user is asked to send photographs of the nutrition information panel (NIP), the list of ingredients, and the front of the package via the crowdsourcing function integrated in the HealthyFood Switch app. Crowdsourcing occurred at a national level, not only in Johannesburg.

### 2.2. Data Entry

Product images, whether collected by in-store surveys or crowdsourcing, are sent to a central electronic holding area where a group of trained researchers then enter the nutrient data into the HealthyFood Switch database. Data entry and quality checking protocols have been described previously [16]. The current database holds records on ~15,000 food products entered between June 2015 and August 2016. Information on energy, total fat, saturated fat, total carbohydrate, sugars, fibre, protein, and sodium levels of foods are virtually complete as they are required to be declared on all food labels in South Africa. For the present study, only food products with nutritional information, including sodium, presented per 100 g (or per 100 mL) on the package NIP were included. Of these, ~85% of packages had nutritional information per 100 g of product “as sold”, the remaining 15% also, or exclusively, reported nutritional information per 100 g of the product, “as prepared”. Foods without a NIP or with multiple NIPs (e.g., variety packs) were excluded. In case of exact duplicates, the most recently entered product was used. The data were cross-sectional and reformulations of foods could not be evaluated. 

### 2.3. Definition of Food Categories

Classification of products followed the food categorisation system of the Global Food Monitoring Group; a standardized system set up to systematically and transparently assess the nutrient composition of processed foods around the world [19]. This hierarchical system classifies foods into groups (e.g., bread), categories (e.g., flat bread), and subcategories (e.g., pita bread), thereby allowing for international comparisons of foods at the group level, while leaving flexibility at the category and subcategory level. The South African HealthyFood Switch database categorisation system contains 15 food groups, 57 food categories, and up to three additional levels of increasingly more specific subcategories. For example, pork sausages are classified in the food group ‘meat and meat products’, food category ‘processed meat’, level 1 subcategory ‘sausages and hotdogs’, level 2 subcategory ‘sausages’, and level 3 subcategory ‘pork sausages’. Foods targeted by the South African sodium legislation were identified by mapping the applicable food subcategories to the categories set out in the legislation. A list of the targeted foods and sodium allowances is provided in Table 1. 

### 2.4. Statistical Analyses

Summary statistics of the sodium levels per 100 g were obtained for each food category, and separately for each food group targeted by the sodium legislation. Medians are reported in the text as these are least affected by extreme large or small values and may give more robust ‘typical’ values. The percentage of targeted foods that met the legislated limits and the amount and percentage by which sodium limits were exceeded were also calculated. For some food groups and categories, only a subset of all foods within that category are targeted by the sodium legislation, that is, the sodium legislation targets a subset of meats and only dry (i.e., powdered) mixes for soups, sauces, stocks, and gravy. For these food categories, we also obtained the summary sodium levels for the individual subcategories. All analyses were carried out in R version 3.3.0 (R Foundation for Statistical Computing, Vienna, Austria). 

## 3. Results

After removing duplicates and products with ineligible or insufficient information on nutritional composition on the NIPs, 11,065 foods were included in the analyses. Of these, 20% were beverages, 16% were processed fruits and vegetable products, 10% were sauces and spreads, 9% were dairy products, 8% were cereal and cereal products, 8% were bread and bakery products, 6% were confectionery, 5% were convenience foods, 5% were meat or meat products, 3% were fish and fish products, and 3% were snack foods. 

### 3.1. Median Sodium Level

There was substantial variation in the sodium level of processed foods within and between food categories (Table A1). The food groups with the highest median sodium level were snack foods (746 mg/100 g), followed by meat and meat products (734 mg/100 g), and sauces and spreads (673 mg/100 g). Cereal and cereal products (70 mg/100 g), fruit and vegetable products (22 mg/100 g), confectionery (66 mg/100 g), and dairy (50 mg/100 g) had relatively lower median sodium levels. Within food groups, food categories with the highest median sodium levels were soups (2017 mg/100 g), sauces (999 mg/100 g), meal kits (939 mg/100 g), cheeses (554 mg/100 g), breads (476 mg/100 g), and noodles (470 mg/100 g). Food categories with the lowest sodium levels included several cereal products (e.g., pasta, maize, rice, couscous; all <10 mg/100 g) and dairy products, excluding cheeses (all <100 mg/100 g).

### 3.2. Sodium Levels of Foods Targeted by the Sodium Legislation

**Figure 1:**
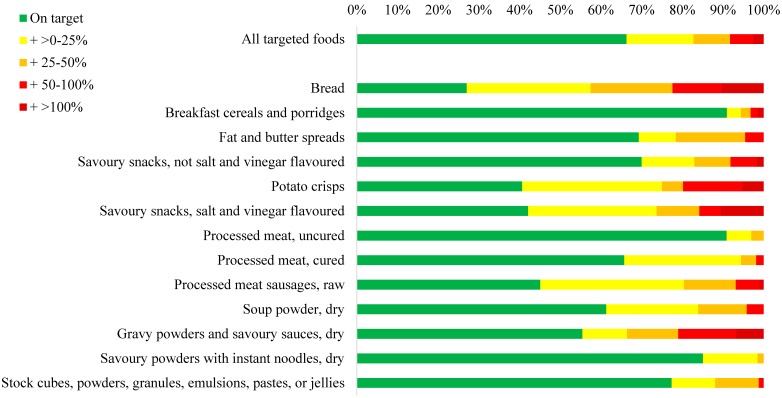
Foods targeted by the sodium legislation according to 2016 sodium limits. Region shaded in green is for foods with sodium levels at or below the sodium limit. The regions shaded in yellow, orange, red, and dark red are for foods with sodium levels 0%–25%, 25%–50%, 50%–100%, or more than 100% above the sodium limit. The maximum total sodium levels allowed in food categories covered by the sodium legislation are given in Table 1. Current sodium levels for targeted foods are provided in Table 2.

The median sodium level of foods targeted by the sodium legislation ranged from 171 mg/100 g for breakfast cereals and porridges to 4782 mg/100 g for dry soup powders (Table 2). Other targeted food groups with very high median sodium levels (i.e., >1000 mg/100 g) were stock (3075 mg/100 g), gravy powders and savoury sauces (3029 mg/100 g), instant savoury powders with noodles (1123 mg/100 g), and salt and vinegar flavoured snacks (1094 mg/100 g). Overall, 67% of all targeted foods had a sodium level below the legislated maximum (Figure 1). Categories with less than 50% of all products achieving the legislated maximum sodium level were bread (27%), potato crisps (41%), salt and vinegar flavoured snacks (42%), and raw processed sausages (45%) (Figure 1). Over 90% of breakfast cereals and porridges and uncured processed meats had sodium levels below the legislated maximum allowed.

### 3.3. Sodium Reductions Needed to Meet the Sodium Target

Of targeted foods exceeding the legislated limits, sodium levels would need to be reduced by a quarter or less for 49% of these foods, by 25%–50% for 26% of foods, by 50%–100% for 17% of foods, and by more than 100% for 7% of foods (Figure 1 and Table A2). In absolute terms, the median reductions in sodium levels required to meet the limits were 110 mg/100 g for breads, 136 mg/100 g for potato crisps, 236 mg/100 g for salt and vinegar flavoured snacks, and 108 mg/100 g for raw processed sausages. Almost 50% of all gravy powders and savoury sauces exceeding the sodium limit, did so by 50% of the limit or more, equating to a median excess sodium level of 1700 mg/100 g. 

### 3.4. Sodium Levels within Categories Partially Targeted by the Sodium Legislation

**Figure 2:**
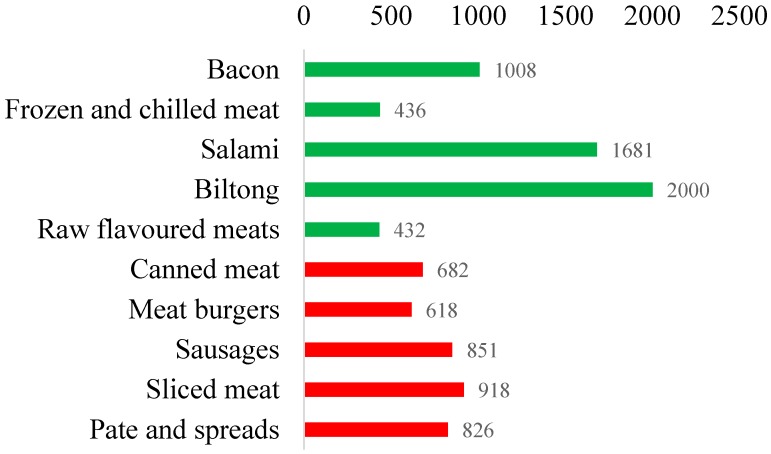
Mean sodium levels of processed meat subcategories in mg per 100 g. Green bars represent meat categories not targeted by the sodium legislation. Red bars represent meat categories targeted by the sodium legislation.

The sodium legislation only targets a subset of meats and only dry (i.e., powdered) mixes for soups, sauces, stocks, and gravy. The median sodium levels of meat products targeted by the legislation was 638 mg/100 g for uncured processed meats, 864 mg/100 g for cured processed meats, and 826 mg/100 g for raw processed sausages. Sodium levels were higher in meats not targeted by the legislation; bacon, salami, and biltong, had a median sodium level of 1070 mg/100 g, 1674 mg/100 g, and 2079 mg/100 g, respectively (Figure 2 and Table A3). Canned and chilled soups, also not targeted by the legislation, had median sodium levels of 373 mg/100 g, and 303 mg/100 g, respectively. Stocks and gravy sold as liquid contained a median of 4000 mg and 429 mg of sodium per 100 g, respectively. Sauces not covered by the legislation that were high in sodium were curry pastes (2400 mg/100 g), Asian sauces (2499 mg/100 g), mustard (1760 mg/100 g), and table sauces (988 mg/100 g) (Table 3).

## 4. Discussion

South Africa is the first country to adopt mandatory legislation for the reduction of sodium levels across a wide range of processed foods. Findings from this study indicate that two-thirds of targeted food items already met the maximum sodium limits during early stages of policy implementation. However, there was variation in the percentage of foods on target across legislated categories; while over 90% of breakfast cereals and uncured processed meats met the sodium targets, just over 40% of all crisps, salt and vinegar flavoured snacks, and raw processed sausages, and fewer than 30% of breads contained less sodium than the current maximum sodium limit. 

Reduction of sodium intake is a global health priority. In 2014, 75 countries representing all WHO regions had national sodium reduction strategies, include food reformulation (81% of countries), front of package labelling (41%), consumer education (95%), and initiatives in public institutions [7,8]. Targets for food reformulation are often voluntary and, in most countries, are only for bread, which is often a large contributor to dietary sodium from processed foods [7]. South Africa, and now also Argentina, are currently the only two countries with mandatory sodium limits for a range of food products across several different food industries. Several other countries have been successful in developing partnerships with the food industry to negotiate voluntary sodium reduction targets for processed foods [9,20]. In the UK, these voluntary sodium reduction targets have led to an estimated 7% decrease in the sodium levels in processed foods and there has been an 8 to 10% decrease in mean population salt consumption between 2006 and 2011 [21,22]. More challenging voluntary sodium targets were set for 2017 in order to achieve further reductions [23,24]. It will be important for the South African government to ensure that the regulated sodium limits are updated regularly to reflect the levels in the current food supply and global best practice. It will also be important to periodically check that the scope of the regulation is adequately capturing all products important to dietary salt consumption in the country.

The ultimate impact of the sodium legislation will be measured by its effect on reducing the burden of cardiovascular disease and associated health care expenditures. A modelling study that informed the development of the sodium legislation in South Africa estimated that a reduction of daily sodium intake of 0.85 g per person per day could avert 7400 cardiovascular deaths; 6400 of which would be due to reducing the sodium levels of bread alone [14]. The additional 4300 non-fatal strokes that could be prevented are projected to save the strained South African health care system 40 million USD a year. An extended cost effectiveness analysis supported these findings and reported that the South African population salt reduction programme could also avert poverty and reduce household out of pocket expenditures, particularly for the middle class, at minimal cost [15]. The impact of the sodium legislation on the burden of cardiovascular disease in South Africa will only become apparent some years after it is implemented. To attribute change in the burden of cardiovascular disease to the sodium legislation, assessment of each step between policy implementation and the anticipated health outcomes is needed, including evaluation of its impact on changes in the sodium levels of foods, population salt intake, and blood pressure levels [25,26,27]. The HealthyFood Switch technologies used in this study provide an objective, practical, transferable, and scalable approach to assess the nutritional composition of packaged foods, to assess whether targeted food products comply with the legislation, and to facilitate global benchmarking. 

This study has some limitations. First, the HealthyFood Switch database mainly comprises foods available from large retailers that predominantly serve the middle to higher socioeconomic urban population. While additional food items were added through crowdsourcing, our data are not necessarily representative of all packaged foods in South Africa. Second, we evaluated the sodium levels of foods available in-store and did not examine actual food purchases or consumption, nor market share of brands. However, there are data from the UK indicating that crude mean sodium levels of product ranges are broadly comparable to the weighted mean sodium levels of products actually sold [28]. Third, since nutritional data were collected between the notification and early implementation period of the sodium legislation, we were unable to determine whether food manufacturers had already commenced reformulating, withdrawing, or replacing high-sodium products before the legislation came into effect. Fourth, sodium levels collected were derived from NIPs of packaged foods, which, although mostly deemed to be accurate [29], are not necessarily derived from chemical analyses. Fifth, in some cases, the availability of ‘as prepared’ nutrition values alone (<15% of products) limited the capacity for robust comparison because mean sodium levels can be influenced by the recommended method of preparation for which there no agreed standards. 

In conclusion, sodium levels of two-thirds of foods covered by the sodium legislation in South Africa already met the sodium target during early stages of policy implementation. Further, only moderate reductions in sodium content will be required to bring many of the currently products in line with the regulation. This represents an excellent opportunity for the South African food industry to make rapid improvements to the national food supply. The high sodium levels of nearly three-quarters of breads, the main contributor to non-discretionary sodium intake in South Africa, will require particular attention and should be an early focus of activity. Continued monitoring of sodium levels in foods is required to support industry action and ensure compliance with the legislation is achieved. Monitoring data will also enable modelled evaluation of the impact of the sodium legislation on dietary sodium intake and its downstream effects on population blood pressure levels and cardiovascular diseases.

## Figures and Tables

**Table 1:** Maximum total sodium levels allowed in certain foodstuffs in South Africa as at June 2016 and June 2019.

Foodstuff Category	Maximum Total Sodium per 100 g per June 2016, Mg	Maximum Total Sodium per 100 g per June 2019, Mg
Bread	400	380
Breakfast cereals and porridges	500	400
Fat and butter spreads	550	450
Savoury snacks, not salt and vinegar flavoured	800	700
Potato crisps	650	550
Savoury snacks, salt and vinegar flavoured	1000	850
Processed meat, uncured	850	650
Processed meat, cured	950	850
Processed meat sausages, raw	800	600
Soup powder, dry	5500	3500
Gravy powders and savoury sauces, dry	3500	1500
Savoury powders with instant noodles, dry	1500	800
Stock cubes, powders, granules, emulsions, pastes, or jellies	18,000	13,000

**Table 2:** Sodium levels of soups, stocks, gravies and sauces (*n* = 962), in mg per 100 g.

Foodstuff Category	No. of Products	Minimum	25%	Median	Mean	75%	Maximum
Bread	174	39	388	476	542	593	2470
Breakfast cereals and porridges	376	0	46	171	262	346	4180
Fat and butter spreads	88	0	339	400	428	625	826
Savoury snacks, not salt and vinegar flavoured	417	0	42	480	519	857	2296
Potato crisps	96	175	554	702	721	802	1670
Savoury snacks, salt and vinegar flavoured	19	510	807	1094	1173	1258	2851
Processed meat, uncured	33	44	500	638	618	784	1065
Processed meat, cured	108	0	656	864	836	998	1667
Processed meat sausages, raw	102	426	708	826	851	914	2213
Soup powder, dry	168	123	2842	4782	4505	6366	9180
Gravy powders and savoury sauces, dry	119	186	500	3029	3197	4997	10,960
Savoury powders with instant noodles, dry	67	1	313	1123	887	1314	1876
Stock cubes, powders, granules, emulsions, pastes, or jellies	84	217	1252	3075	9122	17,270	27,010

**Table 3:** Sodium levels of processed foods in South Africa targeted by the sodium legislation (*n* = 1851), in mg per 100 g.

Food Subcategory		Targeted by Sodium Legislation	No. of Products	Minimum	25%	Median	Mean	75%	Maximum
Soups									
	Dry soup mixes	Yes	164	123	2997	4850	4604	6400	9180
	Diet soup mixes	Yes	4	312	345	356	442	454	746
	Canned soup	No	55	170	260	373	352	418	574
	Chilled soup	No	51	1	262	303	328	398	874
Stocks and gravy									
	Gravy powders	Yes	30	320	1042	3804	3677	5034	10,960
	Stock powders	Yes	36	578	14,780	20,180	18,230	22,810	27,010
	Stock liquids	No	21	458	828	4000	4614	8200	9200
	Gravy liquids	No	5	429	429	429	447	464	484
Sauces									
	Powdered meal-based sauces	Yes	89	186	473	2524	3036	4979	10,600
	Marinades	Yes	48	217	1091	1353	2292	1646	11,250
	Ambient meal-based sauces	No	66	128	422	563	1423	958	8700
	Curry pastes	No	37	47	1217	2400	2597	4000	5770
	Liquid meal-based sauces	No	61	0	425	538	1043	806	8100
	Asian Sauces	No	49	2	991	2499	3229	5752	9640
	Meat accompaniment	No	15	0	12	69	298	353	1770
	Mustard	No	23	423	1230	1760	1959	2300	5500
	Pasta sauces	No	81	57	438	556	651	710	2050
	Table sauces	No	108	0	574	988	1136	1355	5152
	Other sauces	No	19	314	474	703	716	899	1634

## Appendix A

**Table A1:** Sodium levels of packaged foods by food category in South Africa (*n* = 11,065), in mg per 100 g.

Food Group/Category			No. of Products	Minimum	25%	Median	Mean	75%	Maximum
Beverages			2163	0	0	0	31	11	1260
		Fruit and vegetable juices	426	0	3	6	18	13	1205
		Soft drinks	257	0	2	7	14	13	200
		Cordials	113	0	18	56	92	106	667
		Coffee and tea	428	0	0	0	73	20	784
		Electrolyte drinks	38	0	31	42	192	183	1260
		Alcoholic beverages	671	0	0	0	0	0	9
		Waters	144	0	0	2	8	9	100
		Energy drinks	53	0	7	35	33	56	83
		Beverage mixes	18	0	17	205	211	336	667
Bread and bakery products			847	0	250	400	440	582	2827
		Bread	174	39	388	476	542	593	2470
		Biscuits	526	0	222	378	431	614	2827
		Cakes, muffins & pastry	147	20	242	341	353	436	1270
Cereal and cereal products			939	0	6	70	239	296	4180
		Cereal bars	78	0	64	168	178	253	850
		Noodles	78	0	201	470	737	1314	1876
		Breakfast cereals	376	0	46	171	262	346	4180
		Pasta	153	0	2	4	78	20	1440
		Maize (corn)	41	0	3	5	27	16	193
		Rice	64	0	3	8	139	178	1440
		Couscous	18	0	3	10	284	532	1262
		Unprocessed cereals	131	0	3	9	206	65	3710
Confectionery			645	0	22	66	85	108	1380
		Chocolate and sweets	541	0	35	74	95	114	1380
		Jelly	49	0	15	26	31	27	93
		Chewing gum	44	0	0	1	41	13	616
		Cough drops/throat lozengers	11	0	0	0	5	1	49
Convenience foods			586	1	309	442	1624	1887	9180
		Pizza	33	377	435	478	477	513	598
		Soup	270	1	355	2017	2930	5410	9180
		Ready meals	156	12	290	382	422	488	2280
		Pre-prepared salads and sandwiches	82	7	240	303	325	454	818
		Meal kits	43	103	554	939	1198	1678	4700
		Others	2	329	358	386	386	415	444
Dairy			986	0	39	50	209	270	1820
		Cheese	240	0	377	554	654	808	1820
		Yoghurt products	339	0	36	43	47	50	514
		Milk	253	0	38	48	73	55	822
		Cream	30	0	29	36	37	44	142
		Desserts	69	0	61	99	154	266	601
		Ice cream and edible ices	55	0	18	50	49	78	179
Edible oils and oil emulsions			237	0	0	2	169	390	1706
		Butter and margarine	88	0	339	400	428	625	826
		Cooking oils	118	0	0	0	2	1	37
All egg products			46	0	126	126	113	131	196
Fish and fish products			284	0	236	328	384	449	4430
		Canned fish and seafood	144	0	248	321	387	400	4430
		Chilled fish	25	0	162	470	640	876	1620
		Frozen fish	94	38	186	284	297	413	670
		Other fish products	21	223	359	449	456	502	773
Foods for specific dietary use			320	0	15	102	177	260	2050
		Baby foods	203	0	6	27	75	150	306
		Meal replacements	117	0	167	347	354	460	2050
Fruit and vegetable products			1815	0	3	22	509	249	38,800
		Vegetables	895	0	7	108	288	360	3860
		Fruit	466	0	2	8	68	36	3927
		Jam and spreads	86	0	6	10	19	24	151
		Nuts and seeds	166	0	6	22	123	146	1117
		Herbs and spices	202	0	0	0	3028	2444	38,800
Meat and meat products			545	0	464	734	850	1020	4136
		Processed meat and derivatives	486	0	477	732	808	1010	3036
		Meat alternatives	59	1	359	748	1204	1578	4136
		Snack foods	367	0	562	746	785	1020	2851
Sauces and spreads			1059	0	391	673	1981	1634	27,010
		Sauces	704	0	482	999	2700	2818	27,010
		Mayonnaise/dressings	183	0	311	542	581	805	4500
	Spreads		172	0	160	386	531	607	5380

**Table A2:** Sodium levels of foods covered by the sodium regulation containing higher levels of sodium than the maximum allowed.

	On Target, %	Excess Sodium Level, %				Excess Sodium Level, mg/100 g			
		0%–25%	25%–50%	50%–100%	>100%	25%	Median	Mean	75%
Bread	27	30	20	12	10	68	110	229	225
Breakfast cereals and porridges	91	3	2	2	1	96	148	606	382
Fat and butter spreads	69	9	17	5	0	112	150	161	210
Savoury snacks, not salt and vinegar flavoured	70	13	9	6	2	98	240	289	400
Potato crisps	41	34	5	15	5	71	136	248	377
Savoury snacks, salt and vinegar flavoured	42	32	11	5	11	160	236	487	585
Processed meat, uncured	91	6	3	0	0	24	37	87	126
Processed meat, cured	66	29	4	2	0	48	70	149	201
Processed meat sausages, raw	45	35	13	6	1	75	108	192	276
Soup powder, dry	61	23	12	4	0	553	1184	1385	2192
Gravy powders and savoury sauces, dry	55	11	13	14	7	926	1700	2018	2902
Savoury powders with instant noodles, dry	85	13	1	0	0	26	65	128	181
Stock cubes, powders, granules, emulsions, pastes, or jellies	77	11	11	1	0	3587	4631	5384	8037
Total	67	16	9	6	2	86	211	684	516

**Table A3:** Sodium levels of packaged meats in South Africa (*n* = 440), in mg per 100 g.

Meat Type	Targeted by Sodium Legislation	No. of Products	Minimum	25%	Median	Mean	75%	Maximum
Bacon	No	22	552	784	1070	1008	1156	1540
Frozen and chilled meat	No	103	39	336	461	436	548	1080
Salami	No	26	1164	1505	1674	1681	1884	2462
Biltong	No	37	975	1763	2079	2000	2231	3036
Raw flavoured meats	No	16	4	315	428	432	497	1080
Canned meat	Yes	25	0	560	657	682	866	974
Meat burgers	Yes	33	44	500	638	618	784	1065
Sausages	Yes	102	426	708	826	851	914	2213
Sliced meat	Yes	70	387	758	942	918	1020	1667
Pate and spreads	Yes	6	550	816	860	826	865	1020
